# Older Adults and Community-Based Organizations as Research Partners: Three Methodological Case Examples

**DOI:** 10.1093/geroni/igaf122.428

**Published:** 2025-12-31

**Authors:** Michelle Putnam

## Abstract

This session presents approaches to engaging in community-based research using case examples from three individual investigators and their work. The first example, presented by Dr. Susan Stark, describes a trajectory of starting with building community partnerships, then developing participant advisory boards, and finally creating and supporting an ongoing community-based research network of community-based organizations regularly engaged in a collaborative process supporting local research ranging from fall prevention to community participation in greater St. Louis and the Missouri state area. The second example, presented by Dr. Caitlin Coyle, describes a laddered model of building capacity among older adults starting with lower levels stakeholder engagement through to paid, trained membership in the academic research team employed to evaluate age-friendly communities in New England that reflects the needs and preferences of older community members. The third example, presented by Dr. Lydia Ogden, describes a process of building trust between the researcher and study participant in ethnographic research at a clubhouse for older adults with serious mental illness by developing and sustaining a culture of belonging within the collaborative research process that honors participant voices across the study design, including co-authorship of study findings. Shared elements across case examples are identified and discussed, with the aim of facilitating a conversation with the audience about learnings from each model and recommendations for researchers seeking to engage older adults and local communities meaningfully and in a sustained way in their research.

